# Effects and Anti-rotation Stabilization of the Non-bridging External Fixation for Pronation-Abduction Stage III Ankle Fracture: A Cadaveric Study

**DOI:** 10.1155/2021/9966344

**Published:** 2021-05-08

**Authors:** Yili Chen, Xiaoyu Huang, Yongzhong Cheng, Jingjing Xu, Yang Chen, Qi Zhang, Jianmin Wen

**Affiliations:** Wangjing Hospital of China Academy of Chinese Medical Sciences, Beijing 100102, China

## Abstract

**Objective:**

This study is aimed at providing a nonbridging external fixation technique with pinning fixation for the pronation-abduction stage III ankle fracture. The secondary purpose was to evaluate its effect on anatomic reduction and fracture fragment stability against cadaveric models' rotation.

**Method:**

A paired design study was conducted using 14 pairs of the cadaveric model which had been modeled for pronation-abduction stage III ankle fracture. One fracture model from each pair was randomly allocated to receive an open reduction and internal fixation, while the other was reduced and stabilized with the external fixation technique. After the surgery, the antirotational stability tests were performed with external rotation torques of 10 nm, 15 nm, and 20 nm applied, respectively. The postoperation reduction rate and ankle parameters were recorded in anteroposterior and lateral radiographs before and after the antirotational stability experiment.

**Result:**

The outcomes were assessed according to Burwell-Charnley's radiographic criteria of reduction. It showed no statistically significant differences in reduction between the two groups (*P* < 0.05). The displacement of lateral fragment following a reduction in the external fixation group was significantly larger than that of the internal fixation group (3.14 ± 0.56 vs. 1.49 ± 0.39, *P* < 0.05). After applying rotational torques of 10 nm, 15 nm, and 20 nm, the results of other parameters showed no significant differences between the two groups.

**Conclusion:**

This nonbridging external fixation method with pin fixation of fracture fragments might have the same effect as that of internal fixation on the reduction rate of pronation-abduction stage III ankle fracture.

## 1. Introduction

Ankle fracture is a common injury with an annual incidence of 71 to 169/100,000 (0.071%-0.169%) in recent years [1–3]. According to the Lauge-Hansen classification [4], the stages of pronation-abduction (PAB) fracture include the fracture of medial malleolus or rupture of the deltoid ligament (stage I), disruption of the syndesmosis or avulsion of posterior malleolus (stage II), and comminute or transverse fibula fracture proximal to the level of tibial plafond (stage III). Although PAB fracture was constituted less than 9% of all ankle fractures [5], the PAB stage III fracture was worth of particular attention and meticulous management because of its instability [6]. Within the AO Foundation/Orthopaedic Trauma Association (AO/OTA) classification [3], the PAB stage III fracture belongs to total articular fracture (type C). And open reduction and internal fixation (ORIF), advocated by AO/OTA, remained one of the primary treatments for this fracture, which had been widely accepted [7]. It could achieve anatomic reduction, stable immobilization, and early movement of ankle joints. However, severe complications have been reported, including wound infection and even postoperative amputation and mortality in patients with diabetes mellitus [8, 9]. This modality is also contraindicated when soft tissue is poor in many elderly patients [10]. Therefore, various minimally invasive approaches have been investigated, including extraperiosteal plating, a long hindfoot nail, the minimally invasive percutaneous plate osteosynthesis (MIPPO), and provisional external fixation [5, 11–14]. McAndrew found that the application of the external fixator might be effective for trimalleolar fracture [15]. Other researchers had also effectively treated trimalleolar fracture using an external fixator with olive wire or an internal implant [16, 17]. It had been discovered that external fixation was beneficial to the maintenance of extremity length and control of swelling [18]. Ilizarov fixation has also been observed with a lower prevalence of adjacent-joint osteoarthritis after arthrodesis in comparison with internal fixation [19]. Nevertheless, a high rate of pin site infection and pin loosening [20] of external fixation was reported. It might lead to malreduction and traumatic arthritis with the incongruence of the tibiotalar joint. External fixation has also been suggested as an alternative minimally invasive technique for an ankle fracture.

They were two kinds of external fixation, the external bridging fixation and the nonbridging external fixation. The external bridging fixation, as a joint-spanning external fixator, could be used as an early application to achieve the initial goal of stability and the respect of soft tissue. Thereby, the time necessary for definitive treatment could be decreased [21]. Still, recurrent ankle pain, swelling, long-term joint stiffness, and functional impairment are long-term sequelae of the bridging external fixation treatment [22]. Moreover, the nonbridging external fixation had been commonly used for distal radius fractures, although it could improve early motion with nonbridging construction. The function outcomes were similar to the external bridging fixation [23]. There were few studies about nonbridging external fixation for the ankle [24].

This study is aimed at demonstrating a nonbridging external fixation technique to treat the PAB stage III ankle fracture to achieve great reduction and stable fixation. And this technique could be used in the ankle fracture in which tibiofibular syndesmosis is stable and nonseparated. The antirotation stabilization was tested on the PAB stage III fracture in cadaveric models, and the reduction would be evaluated by radiographs [25].

## 2. Material and Methods

### 2.1. Modeling

Fourteen pairs (*n* = 28) of fresh-frozen adult lower leg (bilateral) cadaver specimens were provided by the Beijing Society of Anatomical Sciences. All specimens were without any deformity or preexisting osseous pathology. There were 10 (35.71%) male limbs and 18 (64.28%) female limbs. The median age of the donors at the time of death was 48.25 (38 to 65 range). These specimens were kept storing at -10°C and thawed in a cold-water tank five hours before experimentation.

Although the sample size of the cadaveric study is small, according to some previous research, the sample of this study has been larger than the size of Fitzpatrick et al.'s (*n* = 9) and Kritsaneephaiboon et al.'s studies (*n* = 10) [26, 27].

**Figure 1:**
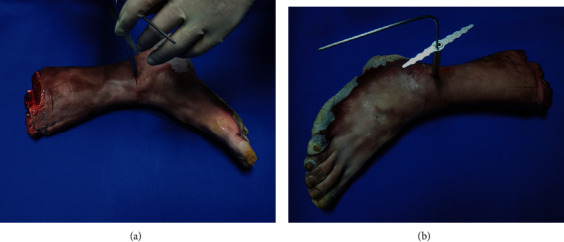
The osteotomy was to draw fracture lines on bone by using the drill bit guider. (a) The medial side. (b) The lateral side.

**Figure 2:**
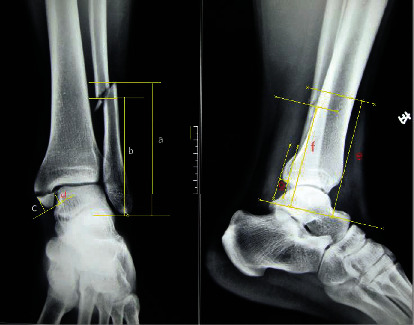
The fracture line of the medial and lateral malleoli obtained from the typical PAB stage III ankle plain radiographs using Digitizer 4.2.6.0. (i) Anteroposterior ankle view: (a) the lateral height of the lateral malleolus; (b) the medial height of the lateral malleolus; (c) the medial height of the medial malleolus; (d) the lateral height of the medial malleolus. (ii) Lateral ankle view: (e, f) the measurement of the lateral malleoli, which could indicate the fracture line trend.

Minimally invasive osteotomy on the specimens first underwent by using the device that majorly contains two parallel cannulas and functions as a drill bit guide (Taizhou Wuyan Medical Technology Development Co., Ltd.). The osteotomy lines (Figure 1) were based on the details of the fracture lines in the medial, lateral, and posterior malleoli obtained from prior measurement of the typical PAB stage III ankle fracture plain radiographs using Digimizer 4.2.6 (Figure 2). The height of the lateral malleolus fracture line was measured on the anteroposterior ankle position, from the fracture fragment end to the tip of the fibula. The lateral malleolus' lateral height was the vertical height of the lateral fragment end of the fibula. The medial height was the vertical height of the medial fragment end. The medial and lateral heights of the medial malleolus were measured as the tibia fracture fragment's vertical height. Then, the fracture line's trend was indicated by the fracture line on the lateral ankle position. Two radiologists measured these values based on 50 radiographs about PAB stage III ankle fracture, which were taken in Wangjing Hospital of China Academy of Chinese Medical Sciences. The medial malleolus fracture lines' lateral and medial lengths were 14.81 (14.84° ± °2.15)°mm and 15.87 (15.87° ± °1.43)°mm. And the lateral and medial lengths of the lateral malleolus fracture lines were 50.35 (50.35° ± °5.22)°mm and 40.35 (40.35° ± °4.17)°mm.

Two surgeons made consecutive holes along the depicted fracture lines. Furthermore, cortical bones were penetrated, as well as the anterior and posterior lower tibiofibular ligament was also released with the drill bit. Other musculotendinous and ligamentous anatomical structures were left intact. During the procedure, X-rays were taken repeatedly to assure quality for each stage by surgeons.

**Figure 3:**
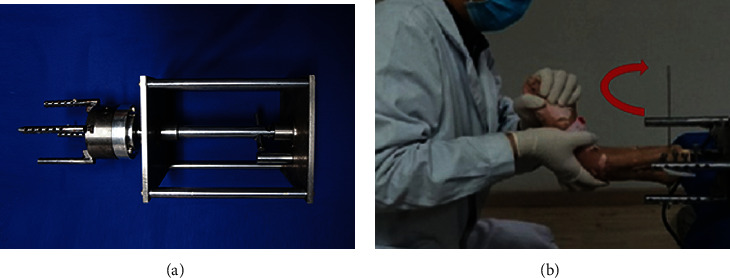
The specimens were rigidly fixed to a mechanical measuring and specimen fixating machine, which was equipped with a force transducer. (a) The mechanical measuring and specimen fixating device. (b) The researcher held the heel with the right hand and held the metatarsophalangeal with the left hand, disposing the ankle in a pronation position and applying abduction force on the talus.

**Figure 4:**
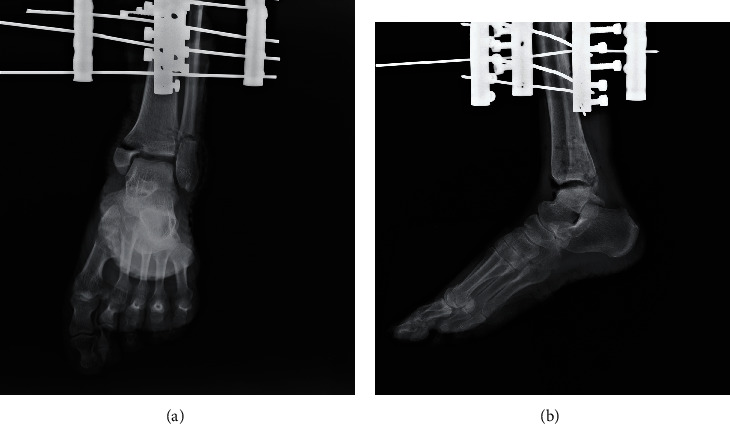
Radiographs were taken after modeling for the verification. A typical model's radiograph. (a) The anteroposterior view. (b) The lateral view.

The specimens were then rigidly fixed to a specimen fixation machine that had a force transducer for the mechanical measuring (Figure 3(a), Taizhou Wuyan Medical Technology Development Co., Ltd.). Next, the rotational forces were exerted by hand to simulate PAB stage III ankle fracture. All procedures were conducted by the same researcher, who held the heel with a right hand and held the metatarsophalangeal with a left hand, disposing the ankle in a pronation position and applying abduction force on the talus (Figure 3(b)). Radiographs were taken for modeling verification by two senior radiologists, shown in Figure 4.

### 2.2. Study Design

A paired design study was conducted. Each left ankle model in a natural pair was randomly assigned into group A (*n* = 14) or group B (*n* = 14) using a random number table, leaving the right ankle allocated into the other group automatically. In group A, specimens were treated with external fixation, while in group B, those were managed with internal fixation. The reductions of two fixation techniques were subsequently assessed by postoperative anteroposterior and lateral radiographs taken immediately. Afterwards, the external rotation experimentation with torques of 10 nm, 15 nm, and 20 nm [28] applied on each ankle was performed, and immediate postreduction radiographic appearance was recorded. The fracture displacement after reduction was evaluated based on Burwell-Charnley's radiographic criteria of reduction [25]. Following the external rotation experimentation, the translation and displacement of the fracture fragments in the two groups were compared.

### 2.3. Reduction and Fixation Technique

#### 2.3.1. External Fixation (EF)

**Figure 5:**
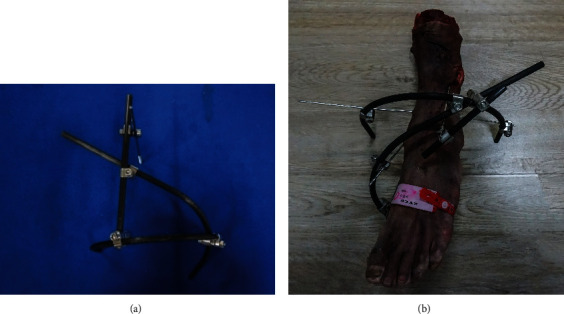
(a) The nonbridging external fixator was constructed by a semicircle rod, an arc-shaped one, and a straight one. (b) The external fixator was installed on a PA stage III ankle fracture.

First, the dislocation of the talus was corrected with manual manipulation. After satisfactory percutaneous clamp reduction, the medial malleolus was fixated by two parallelly inserted 2.0 mm partially threaded olive wires followed by reduction with towel clamps through a small incision. The fibula was intramedullary fixed with the insertion of a 3.0 mm Kirschner wire from the lateral malleolus' apex. It could restore and maintain the fibula alignment. A 2.5 mm olive wire was positioned into the lateral malleolus 2-3 cm proximal to the distal tibial articular surface and through the tibia and fibula. As the subsequent radiographs indicated a satisfactory reduction, a new nonbridging external fixator (Figure 5(a)) would be installed. It was made by a semicircle rod, an arc-shaped one, and a straight one and made of carbon fiber to avoid obscuring fracture lines on radiographs. A 4.5 mm partially threaded half-pin was inserted into a point at the medial tibia (5-7 cm proximal to the distal tibial articular surface) to enhance stabilization. The half-pin penetrated the tibia in an anterior-medial to a posterior-lateral direction at an angle of 15-20 degrees to the tibia's midline (Figure 5(b)).

#### 2.3.2. Internal Fixation (IF)

The lateral approach was taken with a standard 10 cm longitudinal incision made at the lateral fibula. The periosteum was then stripped open with a periosteal stripper. The satisfactory reduction was achieved with reduction forceps under direct visualization. Next, a precontoured 6-hole titanium metaphyseal plate (Synthes) was attached to the lateral fibula's distal aspect. The plate was then stabilized with a 4.0 mm cancellous screw and a 3.5 mm cortical screw distal to the fracture line, and two 3.5 mm cortical screws proximal to the fracture line. The ankle stability was tested intraoperatively via a passive range of motion. An anteromedial approach to the medial malleolus was performed with a curvilinear incision, making approximately transverse fracture line and the interposed periosteum visible. Following careful and acceptable reduction, two 4.0 mm cancellous screws were inserted.

**Figure 6:**
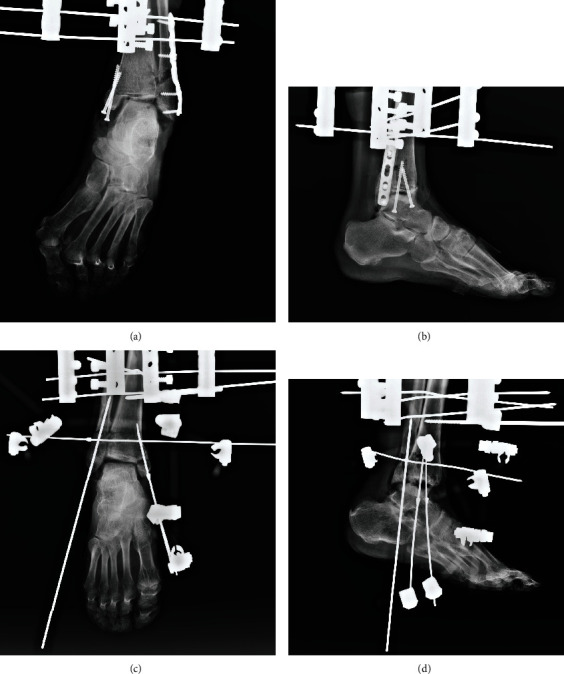
The anteroposterior and lateral radiographs were taken after fixation. (a, b) The internal fixation. (c, d) The nonbridging external fixation.

After fixation, both anteroposterior and lateral ankle radiographs of internal fixation and external fixation were taken for fixation verification, as shown in Figure 6.

### 2.4. External Rotation Test

An external rotation test was performed on these postoperated fracture models in the fixated machine to testify the external fixation technique's stability. The same researcher applied the torsion, and the force was recorded by the transducer connected to the fixation machine. The static 10 nm, 15 nm, and 20 nm external rotational loads were applied. According to the study, fixations could provide failure torques above 20 nm (the failure torque of the screw was 30 ± 9.6 nm) [29]. The ankle's peak joint torques of various activities were well below 20 nm, especially external torsional moments in casts during healing [30]. The intact loading of range of motion was 7.5 nm (20 nm). Consequently, static 10 nm, 15 nm, and 20 nm external rotational loads were applied in this study. After loading, anteroposterior and lateral ankle radiographs of the ankle were taken immediately for further measurement.

### 2.5. Radiograph Measurement and Assessment

According to research, the postoperative ankle radiographs' objective criteria were set out [25]. The reduction was assessed and classified into three levels: anatomic, fair, and poor. Furthermore, the reduction rates of the two fixations were evaluated and compared.

**Figure 7:**
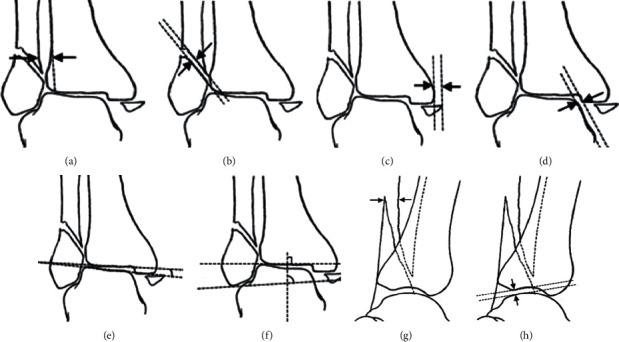
Illustrations of reduction evaluation parameters. AP radiograph: (a) the width of syndesmosis; (b) the lateral displacements (LMD); (c) the displacement of the medial malleolar fragment (MMD); (d) the distance of medial clear space (MCS); (e) talar tilt angle (TTA); (f) talocrural angle (TCA). LAT radiograph: (g) the lateral displacements (LMD); (h) the anterior talar translation (ATT). This picture is partially referenced by the citations [31, 32].

Additionally, as evaluation indexes, parameters were measured on the anteroposterior radiograph (AP) and lateral radiograph (LAT), shown in Figure 7. The width of syndesmosis was measured as the maximum horizontal distance from a point on the tibial incisura one centimetre proximal to the tibial joint surface to the medial part of the cortex of the distal fragment or a line drawn as a proximal extension of the medial part of the cortex [31] (Figure 7(a)). The lateral fragment displacements (LMD) on the AP and LAT were the widest dimension between fracture fragments (Figures 7(b) and 7(g)). The displacement of the medial malleolar fragment (MMD) was the widest dimension on the AP [32] (Figure 7(c)). Medial clear space (MCS) was measured as the distance from the superomedial aspect of the talus to the superomedial corner of the plafond [33] (Figure 7(d)). During the rotation test, the above indexes would be assessed according to the criteria [31].

Meanwhile, to characterize the position of the talus, the talar tilt angle (TTA), the talocrural angle (TCA), and the anterior talar translation (ATT) were observed. TTA was measured as the angle between the line drawn parallel to the distal tibia's articular surface and the line drawn parallel to the talar articular surface (Figure 7(e)). TCA was the angle between the line joining the tip of two malleoli and the line drawn parallel to the articular surface of the distal tibia [34] (Figure 7(f)). ATT was the distance between the posterior lip of the distal tibia and the talar dome [35] (Figure 7(h)).

### 2.6. Statistical Analysis

SPSS® software, version 20.0, was used for statistical analysis. All parameters were analysed with the Shapiro-Wilk test for normal distribution. The chi-square test was used for the comparison of the effectiveness of the two groups. Two groups' evaluation indexes were compared by the paired sample *t*-test. Differences in the external rotation test parameter in the EF groups were assessed by the one-way ANOVA test. Statistical significance was defined at a confidence level of 5% (*P* ≤ 0.05).

## 3. Results

According to Burwell-Charnley's radiographic criteria for fractures, anatomical reduction of this external technique was obtained in 3 cases (21.4%), fair in 10 (71.4%), and poor in 1 (7.14%), with a reduced rate of 92.9%. While in the IF group, 4 cases (28.6%) were evaluated as anatomical and 10 (71.4%) as fair, with a reduced rate of 100%. There was no significant difference in reduction between the two groups (*P* < 0.05, Table 1).

Besides, two fixation techniques showed similar results in tibiofibular syndesmosis, LMD (LAT), MMD, MCS, ATT, TTA, and TCA. There were no significant differences between groups (*P* > 0.05, Table 2). Only the value of LMD (AP) in the EF group was slightly larger than that of the IF group (3.14 ± 2.01 vs. 1.48 ± 1.47, *P* < 0.05). After applying rotational torques of 10 nm, 15 nm, and 20 nm in the EF group, shown in Table 3, these parameters had no significant difference within the group.

## 4. Discussion

According to Lauge-Hansen classification [4], the PAB stage III fracture is biomechanically unstable. Patients can greatly benefit from the open reduction and internal fixation due to its effectiveness in anatomically restoring the ankle mortise. However, this is disputed when skin status is unfit. As a minimally invasive technique, external fixation might be superior in these circumstances. This study showed an innovative pin fixation and nonbridging external fixation technique and evaluated its reduction effectiveness in cadaveric PAB stage III fracture models. Many external fixation concepts have been widely used, and the axial loading of these has been the target of research [36]. Nevertheless, considering the external torsional moments in many activities and the rotational force which caused PAB fracture [30], the antirotation test is also necessary.

This research showed no significant differences between the two groups in tibiofibular syndesmosis, LMD (LAT), MMD, ATT, TTA, and TCA. The LMD (AP) in the EF group was significantly larger (*P* < 0.05). The value was slightly larger due to the poor reduction model in the EF group. Therefore, it might indicate that external fixation cannot pull the lateral fragment anatomically. However, it could reduce the lateral malleolus objectively. Meanwhile, after applying rotational torques of 10 nm, 15 nm, and 20 nm, there were no significant differences within the EF group (*P* > 0.05). The external fixation might have an advantage in the antirotation test. It could maintain the fracture fragment with a stable immobilization.

### 4.1. Distal Tibiofibular Syndesmosis

The distal tibiofibular syndesmosis is a crucial structure of the stable ankle joint. The injury of distal tibiofibular syndesmosis leads to an unstable ankle which can cause the ankle joint's biomechanical dysfunction. It has been reported that the ankle with high fibular fractures (fibular fracture line which was 45 mm above the articular), syndesmosis separation, and positive intraoperative hook test results need a transsyndesmotic pin or screw fixation [37].

In this study, there was no transsyndesmotic screw in the IF groups because the syndesmosis of the models was not separated. In the EF group, a 2.5 mm olive needle was inserted through the tibiofibular bone from the lateral malleolus after reduction. It was above the distal tibial platform and parallel to the distal tibial joint surface. The purpose of this olive needle could strengthen lateral malleolus fixation and pressurization. And it could stabilize the ankle if the syndesmosis is injured or separated.

### 4.2. Medial Malleolus Fixation

When PAB fracture occurred, medial structures became tense and fail foremost. Commonly, the medial malleolus is fractured with or without a rupture of the deltoid ligament. The deltoid ligament maintains the ankle's stability for playing a major role in preventing excessive metatarsal flexion and the external rotation of the ankle [38]. The medial malleolus is vital for providing stability and normal biomechanical function of the ankle joint. Anatomical reduction of medial malleolus fracture can restore medial support, achieve talus consistency, and contribute to lateral malleolus reduction [39]. A malreduction may cause delay of union, nonunion, and traumatic arthritis, which eventually leads to claudication. There have been some reports of the medial malleolus's fixation with unicortical or bicortical screws, sled fixation, tension-band structures, and microchipped t-plates. However, the optimal solution has not been determined [40–43]. Previous research indicates that olive wires could also offer enough compression to maintain the fracture's stability [44–46].

The threaded part of the pin utilized for medial malleolus fixation in this study increased the friction to the bone, which composed a stronger fixation with the combination of compression offered by olive wire. A biomechanical study proved that, by applying olive wire, the control power of shear motion at the fracture site of an oblique fracture could be strengthened [47]. Additionally, the wires were further reinforced by being fixed to the fixator's rings and tensioned [44]. In this study, there was no significant difference between MMD in the comparison of two fixations. Moreover, the MMD result of the external rotation test showed no difference within the EF group. It indicated that the nonbridging external fixation could achieve good reduction and have enough stable immobilization for medial malleolus.

### 4.3. Lateral Malleolus Fixation

According to the research [48], it is widely accepted that fibula reduction aids in the control of talar reliability. Therefore, anatomic reduction of the fibula is crucial for treating the PAB stage III ankle fracture. Malreduction of lateral malleolus would reduce contact areas of the tibiotalar joint and significant growth of the incidence of traumatic arthritis. A previous biomechanical study demonstrated that the fibula's displacements could cause increased contact pressure on the tibiotalar joint surface, including 5 mm of lateral shift, 5 degrees of external rotation, and 2 mm of shortening [49]. Therefore, malreduction or malfixation of lateral malleolus would decrease the stability of the ankle joint. The current standard of treating lateral fibula fracture is the ORIF using plates and screws construct [50]. Some studies have shown that open reduction and internal fixation in high-energy injuries and elderly ankle fractures are associated with a high risk of skin necrosis and infection [51, 52]. There was a report that performed intramedullary fixation on the lateral malleolus. All cases were reduced with an average ankle score of 78% and no infection [53]. Nonetheless, the reaming of the medullary nail could affect the blood supply of the medullary endothelium [54].

Compared with the traditional fixation method, the external fixation technique uses a 3.00 mm Kirschner, which is inserted locally to perform intramedullary fixation for the lateral malleolus. The results showed that LMD (AP) is less favourable in the EF group (*P* < 0.05). But there was no difference in the LMD (LAT) between the two groups. Therefore, it might indicate that external fixation cannot pull the lateral fragment anatomically. The reduction of the lateral malleolus might still be fair. Also, the external fixation of the lateral malleolus had a good antirotation ability in the external rotation test (*P* > 0.05).

### 4.4. Advantages and Disadvantages

External fixation has been reported with a high rate of pin site infection and pin loosening [20, 22], leading to malreduction and traumatic arthritis associated with the incongruence of the tibiotalar joint. Recurrent ankle pain and swelling, long-term joint stiffness, and impaired ankle function are long-term sequelae of the bridging external fixation treatment. However, infection rates are both high in ORIF and internal fixation removal operation in high-energy ankle infection [55–57]. Compared to the traditional internal fixation, the external fixation might cause less damage to periosteum blood circulation and soft tissue to reduce the infection rate [58]. It provides much more protection for the blood supply and fixates the fracture with multiple plane needle fixations with this external fixator offered a strengthened fixation while following the elastic fixation rules at early and middle stages. The external fixation had locking devices in the axial and transverse planes to ensure the multiple planes' stability [59].

Meanwhile, a good load reconstruction could promote the fracture callus quality. And more physiological stress stimulation at the axial and rotational direction is gained [60]. The external fixation is also cost-effective. It has the advantages of simple operation, strong adjustability, and less operation time. Additionally, the carbon fiber fixator can be penetrated by X-ray without blocking local fracture lines to offer a better and more efficient understanding of the reduction situation.

### 4.5. Limitation

Several limitations exist in the study. First of all, the sample size was limited in this study. (*n* = 28). And the models might not be on behalf of the PAB stage III fracture completely. The internal fixation group should be stabilized by a transsyndesmotic pin in further research, even though there was no syndesmosis injury in our specimens. However, it did not affect the comparison results of the reduction rate of the two groups. Meanwhile, due to the lack of muscular control of these specimens, the cadaver's manually applied forces might not be comparable to those experienced in vivo. Despite the results, these indicate that the innovative pin fixation and nonbridging external fixation are effective and stable for PAB fracture. The adverse reactions, which profoundly influence clinical usage, could not be addressed with this investigation. External fixation is universally recognized with the occurrence of pin site infection, nerve or vessel injury, tendon impingement, and/or joint stiffness [61]. As a result, additional clinical studies are necessary to verify further both the efficiency and safety of utilizing pining and nonbridging external fixation for treating PAB stage III fracture. Nevertheless, despite our appreciation of our investigation's limitations, we believe that the results of this study could be useful.

## 5. Conclusion

The application of an external fixator for PAB stage III ankle fractures is useful, effective, and economical. It has an advantage in antirotation biomechanics for the fixation. Its benefits include maintaining the alignment of fracture fragments and the relative immobilization of the ankle joint that creates good conditions for repairing the ligament, bone, and soft tissues.

In conclusion, the study introduces a stable pin fixation-involved nonbridging external fixation technique, which shows effectiveness for reducing the PAB stage III fracture in a cadaver study. This approach might be advantageous in treating the PAB stage III fracture.

## Figures and Tables

**Table 1:** The reduction effectiveness in two groups (*n* = 28).

Group	Anatomical	Fair	Poor	*n*%	
EF	3	10	1	92.86%	*X* ^2^ = 1.14
IF	4	10	0	100%	*P* = 0.57

**Table 2:** The result in two groups (*n* = 28) prior to the test (mean ± SD).

Parameters (mm)	EF	IF
Tibiofibular syndesmosis	3.13 ± 2.06	3.45 ± 1.61
LMD (AP)	3.14 ± 2.01	1.48 ± 1.47^∗^
LMD (LAT)	1.38 ± 1.40	0.94 ± 1.59
MMD	1.27 ± 1.05	1.58 ± 1.89
MCS	3.62 ± 1.22	2.92 ± 0.73
ATT	3.75 ± 1.81	3.63 ± 0.46
TTA (°)	4.72 ± 2.87	4.95 ± 3.03
TCA (°)	78.81 ± 3.98	78.17 ± 6.39

LMD (AP): lateral malleolus displacement measured on the anteroposterior radiograph (AP); LMD (LAT): lateral malleolus displacement measured on the lateral radiograph (LAT); MMD: medial malleolus displacement; MCS: medial clear space; ATT: the anterior talar translation; TTA: talar tilt angle; TCA: talocrural angle. ^∗^Significant difference tested by the aired sample *t*-test, *P* < 0.05.

**Table 3:** The result in the EF groups (*n* = 14) after tests with rotational torques (mean ± SD).

Parameters (mm)	0 nm	10 nm	15 nm	20 nm	*F*	*P*
Tibiofibular syndesmosis	3.13 ± 2.06	3.51 ± 1.73	3.97 ± 1.75	4.62 ± 2.65	1.33	0.28
LMD (AP)	3.14 ± 2.01	4.42 ± 3.01	4.39 ± 3.04	4.16 ± 2.65	0.65	0.59
LMD (LAT)	1.38 ± 1.40	1.65 ± 1.67	2.02 ± 1.87	2.32 ± 1.61	0.88	0.46
MMD	1.27 ± 1.05	1.93 ± 2.12	2.56 ± 2.66	2.49 ± 1.90	1.20	0.32
MCS	3.62 ± 1.22	3.89 ± 1.82	5.69 ± 4.35	6.06 ± 5.22	1.68	0.18
ATT	3.75 ± 1.81	4.20 ± 2.28	4.22 ± 2.83	4.66 ± 2.80	0.31	0.82
TTA (°)	4.72 ± 2.87	4.71 ± 3.20	5.58 ± 3.69	7.80 ± 7.60	1.33	0.27
TCA (°)	78.81 ± 3.98	80.62 ± 5.80	81.15 ± 5.93	81.12 ± 6.71	0.53	0.67

^∗^Significant difference compared under the different torsions in the EF group tested by the one-way ANOVA test, *P* < 0.05.

## Data Availability

The research data was recorded by Wangjing Hospital. It was funded by the Natural Science Foundation of Beijing (No. 84739131) (China). And the data was recorded in it.
